# The promise of organ rejuvenation to overcome the shortage in organ transplantation

**DOI:** 10.1038/s41467-025-66133-9

**Published:** 2025-12-10

**Authors:** Mukhammad Kayumov, Zhuolun Song, Friederike Martin, Sarah Tsou, Yao Xiao, Hao Zhou, Stefan G. Tullius

**Affiliations:** 1 Division of Transplant Surgery and Transplant Surgery Research Laboratory, Brigham and Women’s Hospital, Harvard Medical School, Boston, MA USA; 2 Department of Surgery, CVK | CCM, Charité Universitätsmedizin Berlin, Berlin, Germany

**Keywords:** Translational research, Senescence

## Abstract

Organ shortage remains a major barrier in treating end-stage organ failure, with many patients dying while waiting or becoming medically unfit by the time an organ is offered. A substantial number of organs, particularly from older donors, remain unused due to concerns over age-related decline in quality. This review highlights emerging strategies to rejuvenate and optimize such organs by mitigating ischemia-reperfusion injury and reducing age-related immunogenicity. Advances in organ preservation, perfusion technologies, and novel therapies – including senotherapeutics, anti-inflammatory agents, and stem cell treatments – show promise in improving graft viability and bridging the gap between organ supply and demand.

## Abstract (web summary)

Organs from older donors are often underutilized due to concerns on inferior quality and function. Here, the authors show that emerging rejuvenation strategies, including machine perfusion and senotherapeutics may enhance viability and quality, thus providing novel approaches to expand the donor pool.

## Introduction

Organ transplantation remains a critical and life-saving treatment for patients with end-stage organ failure. However, the growing demand for transplantable organs and the critical shortage of suitable donors continue to be the most significant barriers to providing patients with the desperately needed opportunity for transplantation^1^. This problem is further aggravated by a steady growth of waitlisted patients, with an increase by 12% over the last three years^2^. As of November 2024, more than 104,000 patients have been waiting for an organ in the United States^2^. This stark discrepancy between the need for organs and their availability results in considerable waiting-list morbidity, with around 7000 patients dying annually while awaiting transplantation in the US alone^2^.

**Fig. 1:**
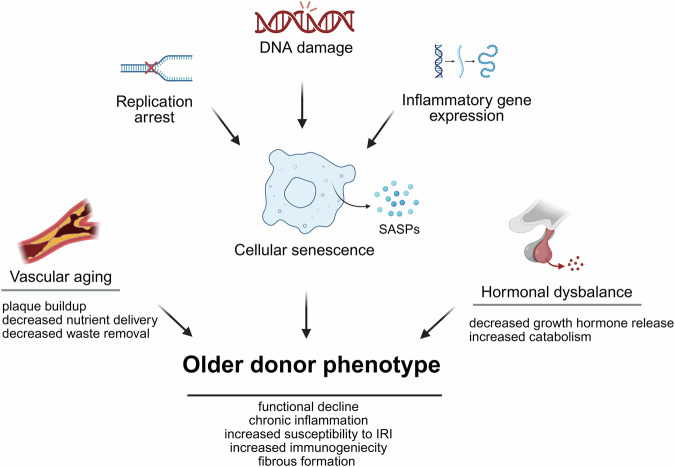
Characteristics of older transplantable organs. Aging results into distinct changes of organ function. Exposure to toxins, UV light, infections, and chronic inflammation contributes to DNA damage, replication arrest, and inflammatory gene expression, collectively driving cellular senescence. Senescent cells accumulate and release pro-inflammatory factors known as the Senescence-Associated Secretory Phenotype (SASP). These secreted factors attract immune cells, induce local inflammation, and disrupt the function of neighboring cells. Over time, persistent inflammation fosters fibrosis, further impairing tissue function. In vascular endothelial cells, cellular senescence contributes to atherosclerotic plaque formation, narrowing the vascular lumen while diminishing nutrient delivery and waste removal in perfused organs. Concurrently, senescent endocrine cells experience a decline in growth hormone secretion, disrupting tissue homeostasis and driving a shift toward catabolism. Together, these cellular and systemic alterations culminate in the hallmark manifestations of aging phenotypes. IRI ischemia reperfusion injury. *Created in BioRender. Kayumov, M. (2025)* https://BioRender.com/m23u7ro.

A major contributing factor to the shortage of usable organs is the under-utilization of organs from older donors. Concerns about quality and outcomes when utilizing older donor organs lead to their frequent discard. It is estimated that nearly 40,000 organs, predominantly from donors over 50 years old, go unused annually^3,4^. This high discard rate has prompted many investigators to focus on improving the utilization of older donor organs. Clinical studies have shown that organs from donors greater than 50 years have a higher probability to be damaged subsequent to prolonged periods of ischemia and reperfusion injury (IRI) with an augmented probability for early graft dysfunction^5–7^. Older organs are also often deemed less suitable due to several age-related impairments, including functional decline, decreased physiological reserves, and compromised repair capacities (Fig. 1)^8–11^. Thus, addressing these age-related challenges may help to optimize the use and suitability of older organs. To reach an optimized utilization, advanced pharmacological interventions, novel perfusion systems and stem cell therapies offer significant promise (Table 1).

**Table 1:** Potential rejuvenative strategies for older donor organs

Interventions	Categories	Agents	Mechanism	References
Pharmacological	Senotherapeutic agents	Dasatinib, Quercetin, Fisetin, Navitoclax, Metformin, Rapamycin, Resveratrol, Ruxolitinib,	Eliminates senescent cells and associated secretome	^47–64^
	Anti-inflammatory agents	Corticosteroids, NSAIDs, VEGF- α, Statins,	Reduces inflammation	^65–73^
	Mitochondrial modulators	Coenzyme Q10, Nicotinamide riboside, Pyrroloquinoline Quinone	Restores mitochondrial function	^74–81^
	Anti-fibrotic agents	Losartan, Blebbistatin, Nattokinase, Lumbrokinase	Reduces fibrosis (fibrinolytic, antifibrotic)	^82–87^
Biotechnological	Stem cell therapy	MSCs, iPSCs	Repairs tissue volume	^88–96^
	Vaccine therapy	GPNMB	Selectively eliminates senescent cells	^97^
Novel organ preservation methods	Organ perfusion	NMP, NRP	Provides nutrients and oxygen during preservation	^98–108^
	Subnormothermic preservation	10 °C storage	Static storage	^109–112^
	Heterochronic transplantation	Transplantation	Old organ to young recipient	^113–120^

*NSAIDs* non-steroid anti-inflammatory drugs, *VEGF-α* vascular endothelial growth factors, *MSCs* mesenchymal stem cells, *iPSCs* induced pluripotent stem cells, *GPNMB* glycoprotein nonmetastatic melanoma protein B, *NMP* normothermic machine perfusion, *NRP* normothermic regional perfusion.

## Utilizing older organs for transplantation

### Ischemia-reperfusion injury

**Fig. 2:**
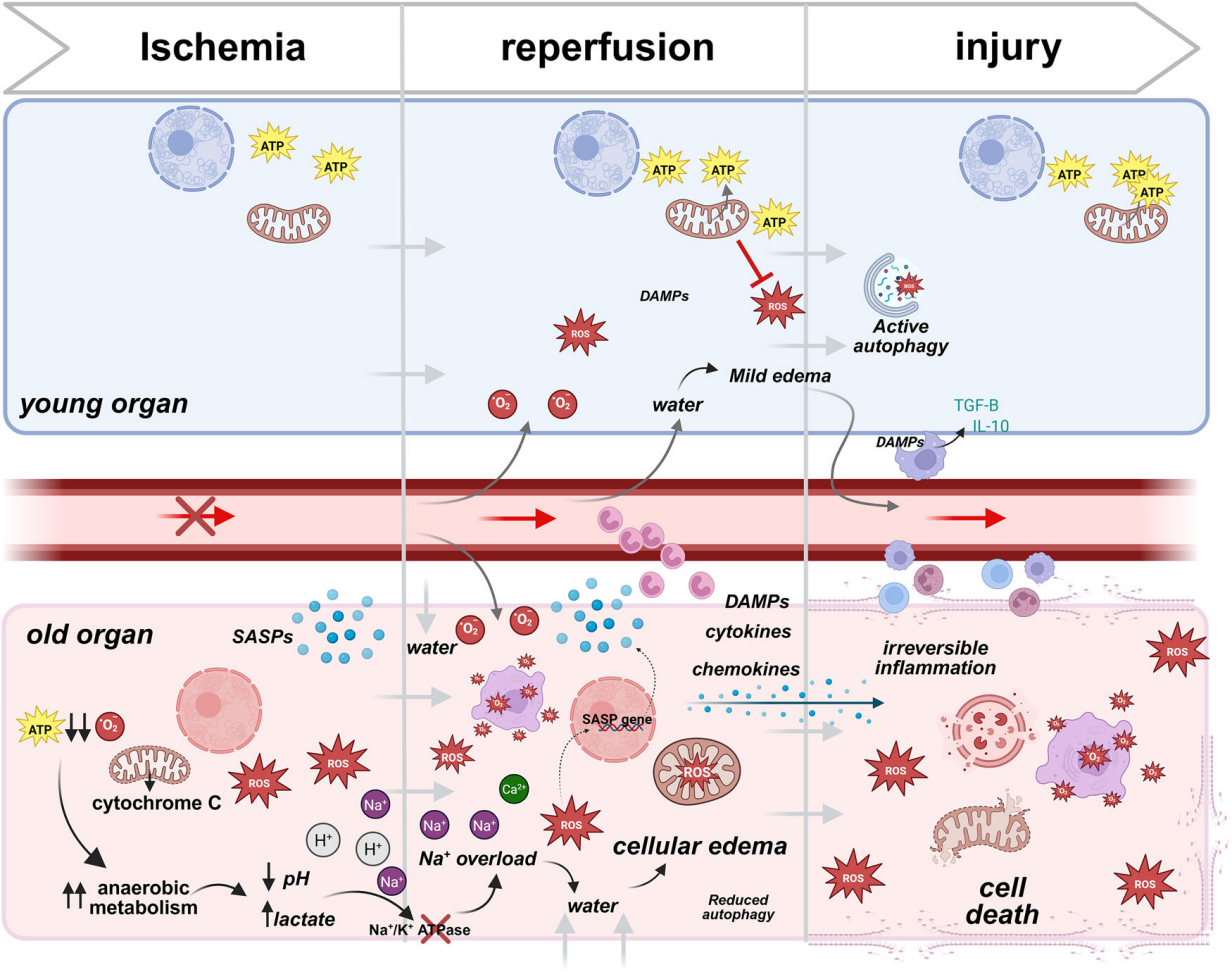
Cellular responses to ischemic reperfusion injury in young vs older donor organs. During ischemia, the deprivation of oxygen and nutrients leads to mitochondrial dysfunction and energy loss. In young cells, mitochondrial resilience helps sustain ATP production, ameliorating damage. Old cells, in contrast, experience significant ATP depletion, relying heavily on anaerobic metabolism, which leads to lactate buildup, pH reduction, and cellular stress. Upon reperfusion, the restoration of blood flow triggers oxidative stress as mitochondria generate excessive reactive oxygen species (ROS). Young cells compensate for those events through robust antioxidant systems, maintaining cellular integrity. Conversely, in old cells, impaired antioxidant defenses result in unregulated ROS production, furthermore damaging membranes, organelles, and DNA. Additionally, old cells release pro-inflammatory genes, amplifying local inflammation. Consequences are particularly severe in aged vascular endothelial cells, with ion pump dysfunction (e.g., Na⁺/K⁺ ATPase) causing ionic imbalances and cellular edema. This disruption exacerbates ischemic injury, progressing to irreversible damage. In contrast, young cells effectively resolve edema and inflammation through mechanisms that include macrophage-mediated clearance of Damage-Associated Molecular Patterns (DAMPs) and anti-inflammatory cytokine release (e.g., IL-10 and TGF-β), allowing recovery and tissue repair. In old cells, persistent ROS generation, unresolved inflammation, and DAMP accumulation lead to irreversible inflammation, organelle collapse, and eventual cell death. *Created in BioRender. Kayumov, M. (2025)* https://BioRender.com/m23u7ro.

Ischemia-reperfusion injury (IRI) represents and integral component of almost all transplantation approaches, characterized by sterile inflammation and oxidative stress initiated during ischemia and exacerbated upon reperfusion^12^. During ischemia, oxygen deprivation reduces ATP production and mitochondrial function, promoting acidosis, ionic imbalance, and cell swelling (Fig. 2)^13^. Upon reperfusion, reoxygenation paradoxically increases reactive oxygen species (ROS), leading to endothelial damage, activation of leukocytes, and release of pro-inflammatory cytokines, which together contribute to tissue injury and primary graft dysfunction (PGD)^14,15^

Current donor organ preservation techniques including MP at differing temperatures, together with donor pretreatments aim to limit the damaging consequences of IRI^16^. However, clinical and experimental evidence demonstrate that older organs are more prone to the consequences of IRI and frequently do not recover after IRI, which can thus limit their utilization (Fig. 2)^16–18^. In fact, an analysis of more than 2000 consecutive liver transplants identified older donor age as an independent risk factor leading to PGD^19^. As further confirmation, liver transplants from donors >50 years demonstrated more extensive ischemic damage on histological analysis immediately after reperfusion^20^. The detrimental correlation between donor age and prolonged preservation time has also been confirmed for renal allografts, with older donor age and prolonged ischemia linked to increased functional and morphological deterioration post-transplantation^21^.

The increased susceptibility to IRI in experimental heart and lung transplantation has been shown to go along with a reduced expression of the MG53 protein—a protein that suppresses NF-kB-mediated inflammation^22,23^. It has been shown that MG53 levels decrease with age, contributing to reduced tissue resilience, increased susceptibility to IRI, and impaired regenerative capacity in older organs^23^. Older livers exhibited augmented microvascular damage due to lower reserves of ATP and glycogen^24^. Experimental studies have also shown that older kidneys are more susceptible to the consequences of ischemia-reperfusion injury, leading to augmented structural damage and heightened immunogenicity due to age-related amplification of oxidative stress and pro-inflammatory immune responses^25,26^. Thus, preventing IRI in older organs has a great potential in improving and optimizing both utilization and outcomes after transplantation.

It is also important to note that the susceptibility to IRI varies across different organs. While the aged heart and lungs are more susceptible to IRI, livers and kidneys tolerate prolonged ischemic times. This variability in IRI susceptibility is largely determined by each organ’s intrinsic regenerative capacity, metabolic demand, baseline functional reserve, and the burden of pre-existing comorbidities.

### Functional decline

One of the key challenges with the utilization of older donor organs for transplantation concerns an age-related functional decline. With age, organs undergo various structural changes that can negatively impact their functionality and overall suitability for transplantation. A key characteristic finding is the increasing accumulation of senescent cells. Senescent cells are characterized by markers that include the lysosomal enzyme senescence-associated β-galactosidase (SA-β-gal), cyclin-dependent kinase inhibitors (notably p21^CIP1^ and p16^INK4a^) and cell free mitochondrial DNA segments the nuclear senescence-associated secretory phenotype (SAHF)^27^. Characteristically, senescent cells accumulate over time, discontinue replication, lose their functionality, and release inflammatory factors collectively known as the senescence-associated secretory phenotype (SASP)^28^. SASP disrupts tissue homeostasis by interfering with the physiological function of neighboring cells, promoting local inflammation. Over time, these processes lead to fibrosis, tissue and organ dysfunction^29^. Recent research has shown promise that eliminating the senescent cells through the application of senolytics—drugs that specifically target and eliminate senescent cells—improves transplant function^28^. Preclinical studies have also demonstrated that senolytics can reduce levels of SASP, diminish cardiac hypertrophy, and improve left ventricle function following transplantation^30^.

Another relevant age-specific aspect is the decline of vascular function^31^. As blood vessels age, they lose elasticity linked to atherosclerosis (plaque buildup)^32^. As a result, blood flow to organs is impaired, reducing the delivery of oxygen and essential nutrients. At the same time, compromised blood flow also hampers the removal of metabolic waste products, further exacerbating organ dysfunction. As an example, it has been well established that the total hepatic blood flow is reduced by 30–50% in individuals >50 years^33^. Moreover, with age, the body’s water content decreases, leading to increased blood viscosity^34^. This higher viscosity contributes to further impaired blood flow and organ perfusion, thus compromising nutrient delivery and waste removal^35^. Age-related changes are also evident in the neuroendocrine system, where the reduced secretion of essential regulatory hormones or a blunted hormonal response disrupts tissue homeostasis, thus augmenting organ dysfunction^36^. Notably, pretreating old donor rats with pooled young plasma has been shown to reduce age-dependent liver IRI while improving graft function, indicating that the systemic environment drives, at least in part, the functional decline of older donor organs^37^.

### Augmented immunogenicity

Aging significantly affects the immunogenicity of donor organs, increasing their susceptibility to immune-mediated damage while augmenting acute rejection rates^38,39^. Our broad clinical analysis of renal transplant recipients from the UNOS database has shown that older grafts are particularly prone to acute rejections, especially within the first-year post-transplant^21^. The increased immunogenicity and higher rejection rates appear, at least in part, related to the compromised reserve and recovery capacity of older grafts^40^. While IRI alone provokes innate immune activation, aged organs experience more severe injury with an augmented release of DAMPs, amplifying immune recognition. At the same time, impaired regenerative and anti-inflammatory responses in older grafts limit their ability to resolve inflammation and recovery from peri-transplant insults. Together, these factors create an amplified pro-inflammatory and immunogenic environment in old organs that contributes to early graft dysfunction and rejection.

Our recent studies found that donor age and IRI synergistically increase DAMP release, with older mice showing a 15-fold increase in cf-mt DNA compared to young controls^30^. The critical role of high-level circulating cf-mt DNA levels has also been confirmed in clinical lung transplantation studies with high rates PGD^41^.

The augmented release of SASP factors is also critically contributing to augmented immunogenicity of older grafts^42,43^. While the composition of SASP can vary based on cell type and stimulus, this process attracts recipient immune cells, induces localized inflammation, thereby increasing the risk of graft rejection^44^. In experimental models, it has been shown that kidneys from older donors provoke a stronger early immune response with a subsequent pronounced functional and structural deterioration, suggesting that advanced donor age not only impacts intrinsic graft function but also intensifies alloimmune reactivity^45^. Experimentally, advanced donor age and prolonged warm ischemia time significantly enhanced alloimmune responses while impairing long-term function in experimental cardiac transplants that had been procured from donors after cardiac death (DCD)^46^. This study emphasizes that older DCD kidneys are more vulnerable to immune-mediated damage, while younger DCD kidneys appear to recover from ischemic injury without sustaining a heightened long-term immune response or damage.

Thus, understanding the molecular underpinnings that drive the augmented immunogenicity of older organs may pave the way for targeted interventions that can mitigate immune-mediated damage, improve graft function, and enhance long-term outcomes in recipients of aged donor organs.

## Pharmacological rejuvenative strategies

### Senotherapeutics

Cellular senescence seems to play a critical role for both onset and progression of age-related complications in older organs, thus eliminating senescent cells offers as a potential strategy to improve graft function and reduce postoperative complications.

**Fig. 3:**
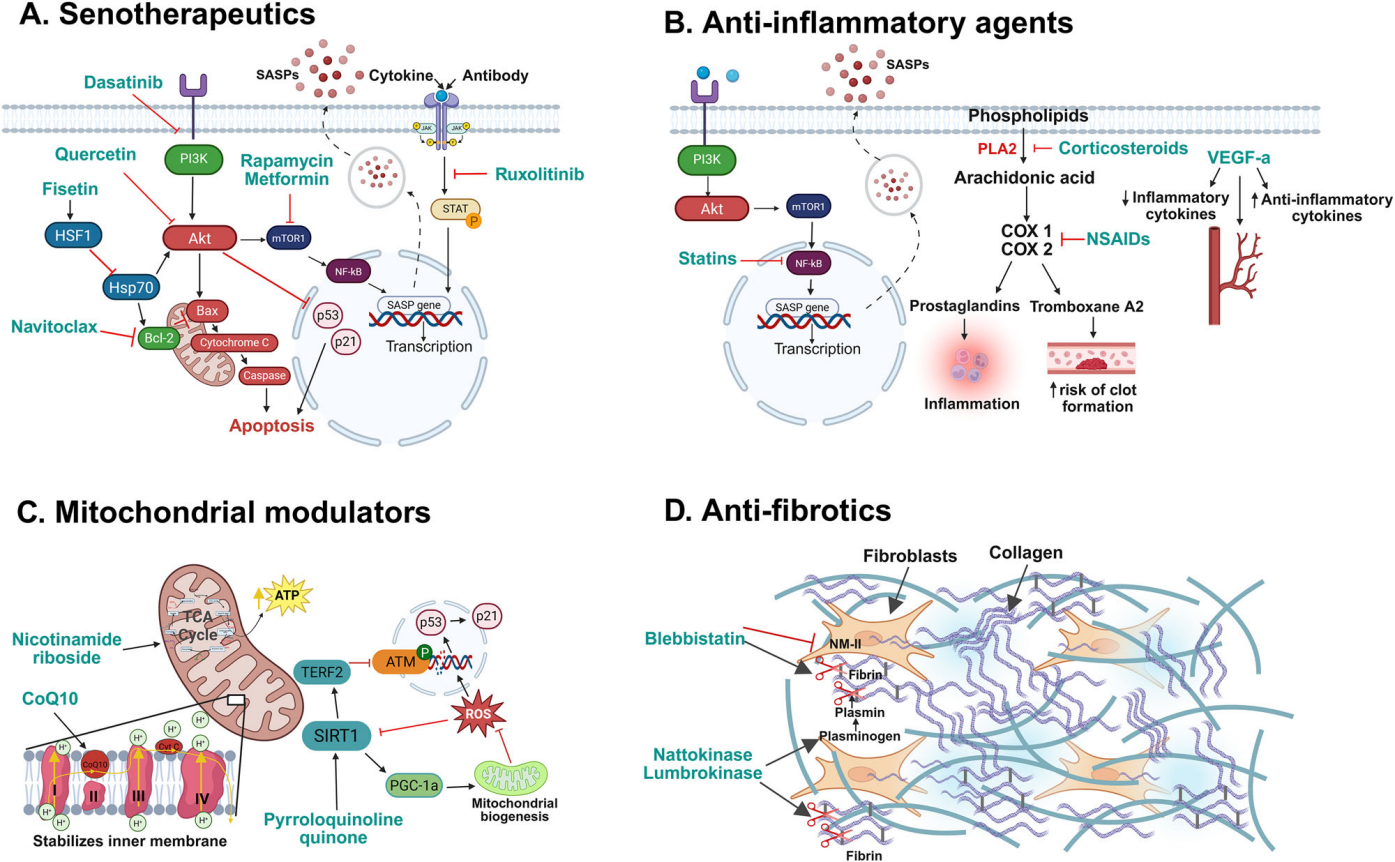
Pharmacological rejuvenative strategies targeting aging-related organ dysfunctions. Various pharmacological strategies may support the rejuvenation of old organs; agents are categorized into four therapeutic approaches: (**A**) senotherapeutics, (**B**) anti-inflammatory agents, (**B**) mitochondrial modulators, and (**D**) anti-fibrotics. *Created in BioRender. Kayumov, M. (2025)* https://BioRender.com/m23u7ro.

Senotherapeutics are a new generation of drugs that focus on two main types of interventions: senolytics, which selectively remove senescent cells, and senomorphics, which interfere with factors of the SASP^47,48^ (Fig. 3A).

#### Senolytics

Since their introduction in 2015, senolytic drugs have been applied to multiple age-related diseases^49^. Eliminating senescent cells is challenging due to their natural resistance to apoptosis, which makes most traditional cytotoxic drugs ineffective. To circumvent this issue, senolytics focus on targeting the Senescent Cell Anti-Apoptotic Pathways (SCAPs) by inhibiting or activating specific proteins that regulate apoptosis resistance^50^. Since senolytics effectively reduce the number of senescent cells and help alleviate age-related dysfunction and inflammation, these agents may play a very relevant role in ‘rejuvenating’ older donor organs.

Dasatinib (D), a broad-spectrum tyrosine kinase inhibitor, and Quercetin (Q), a plant-derived flavonoid that targets BCL-2 family proteins^51^, are among the most well-studied and widely used combinations in clinical trials to eliminate senescent cells. D is a potent inhibitor of Src family kinases (SFKs) and BCR-ABL tyrosine kinase receptors. By inhibiting SFKs, D reduces the phosphorylation of downstream targets including p21 and cyclin D1, leading to the activation of apoptotic pathways. Q and other senolytic flavonoids enhance apoptosis by inhibiting BCL-2 anti-apoptotic family proteins, including BCL-xL, as well as HIF-1α and other components of the SCAP network. Notably, studies have demonstrated that D and Q (D&Q) are most effective when used in combination. Experimental models have shown that D&Q can effectively eliminate senescent cells from various tissues, including adipose tissue, bones, heart, and blood vessels^48^. Our own experimental work has shown that the treatment of old donor heart donors with D&Q significantly reduces the burden of senescent cells, alleviated systemic inflammation, and decreased levels of cf-mt-DNA in the a murine transplantation model^30^.

Fisetin, a natural flavonoid found in various fruits and vegetables, has also shown promising senolytic properties with the selective induction of senescent cell death^52^. Fisetin induces apoptosis by blocking the binding of Heat Shock factor 1 (HSF1) to the hsp70 promoter, thus reducing the expression of heat shock proteins including HSP70 and BAG3. These proteins usually help senescent cells survive by stabilizing anti-apoptotic proteins including Bcl-2. The downregulation of these proteins leads to cellular apoptosis. The senolytic activity of fisetin has been demonstrated in many preclinical studies^53^. Navitoclax (ABT263), another flavonoid senolytic targets and eliminates senescent cells by inducing apoptosis^54^. The drug works by inhibiting the Bcl-2 and Bcl-xL proteins, anti-apoptotic factors that senescent cells rely on to avoid cell death. By blocking these proteins, Navitoclax triggers the death of senescent cells while sparing non-senescent cells. Studies have shown that Navitoclax reduces senescent cells and SASP factors in the myocardium attenuating cardiac hypertrophy and reducing the size of the left ventricle^55^.

#### Senomorphics

Senomorphics are a class of drugs that modulate the effects of senescent cells without killing/depleting them^56^. Senomorphics mainly work by inhibiting SASP factors, including pro-inflammatory factors that can contribute to donor organ dysfunction and immunogenicity after transplantation^57^. Although our understanding on the specific application of senomorphics in transplant models is currently limited, treatment with senomorphics has shown reduced incidences of various age-related diseases while expanding lifespan in animal models.

Metformin, commonly used as a treatment for diabetes, has strong senomorphic capacities^58^. By inhibiting the mTOR pathway, metformin reduces the production of pro-inflammatory cytokines and other components of the SASP. Metformin also activates AMP-activated protein kinase (AMPK), which plays a crucial role for cellular energy homeostasis^59^. AMPK has the capacity to reduce inflammation and oxidative stress, both of which are associated with cellular senescence. Metformin also triggers immune-mediated clearance of senescent cells by modulating the SASP and sirtuin 1-p300-p53-p21 pathway. In-vitro studies have demonstrated that metformin can inhibit SASP factors reducing the secretion of pro-inflammatory cytokines^59^. In the transplant setting, oral metformin given prior to organ retrieval reduced hepatobiliary injury and improved ATP production during normothermic machine perfusion and after orthotopic liver transplantation^60^.

The mTOR inhibitor Rapamycin, also used as an immunosuppressant, also has senomorphic capacities. Rapamycin binds to the intracellular receptor FK506-binding protein 12 (FKBP12), forming a complex that inhibits mTOR complex 1 (mTORC1). This inhibition reduces cell growth and proliferation, protein synthesis, and the production of pro-inflammatory cytokines. In a preclinical kidney transplant model, short-term treatment with low-dose rapamycin during the first 7 days post-transplantation prevented the accumulation of senescent cells, suppressed inflammatory cytokine expression, and helped preserve graft structure and function^61^.

Resveratrol, a natural compound found in grapes, berries, and peanuts, is another potential senomorphic^42^. Research has shown that resveratrol can inhibit the SASP in cultured human cells, thus reducing the secretion of pro-inflammatory cytokines. Experimentally, resveratrol has extended both life- and health span by enhancing mitochondrial function and reducing oxidative stress^62^. Moreover, early-phase clinical trials have reported that resveratrol supplementation improves metabolic parameters including insulin sensitivity while reducing systemic inflammation in older adults^63^. While clinical data in the transplant setting remain limited, these systemic benefits suggest that resveratrol may support improved organ quality and resilience, especially in older donor organs.

Ruxolitinib is a Janus kinase (JAK) inhibitor that has been shown to have senomorphic effects by targeting the JAK/STAT signaling pathway. Studies have shown that Ruxolitinib can protect endothelial function after IRI by reducing the release of cytokines and protecting the endothelium. Moreover, ruxolitinib has been demonstrated to mitigate cardiomyocyte senescence in models of septic cardiomyopathy by inhibiting the JAK2/STAT3 signaling pathway^64^.

### Anti-inflammatory agents

Inflamm-aging, the chronic low-grade inflammation associated with aging, is a major contributor to graft dysfunction in older donor organs^21^. Targeting inflammation and oxidative stress before or during transplantation can thus enhance the quality of older organs while reducing the risk of rejection (Fig. 3B).

Corticosteroids including prednisone and dexamethasone are commonly used in transplantation for their immunosuppressive and anti-inflammatory properties^65^. Corticosteroids exert their anti-inflammatory effects through several mechanisms interfering with the expression of multiple inflammatory genes, including those encoding SASPs and DAMP factors. They further mimic the effect of cortisol, a hormone that naturally suppresses the immune system. Additionally, they also inhibit the production of prostaglandins and leukotrienes which are essential to promote inflammation. So far, corticosteroids are mainly used in recipients to prevent organ rejection by suppressing both innate and adaptive immune responses. However, experimentally, it has been shown that treating organs, especially those from marginal donors, with corticosteroids before transplantation reduces the risk of rejection^66^.

Non-steroidal anti-inflammatory drugs (NSAIDs) such as aspirin, ibuprofen and naproxen represent additional potential candidate to suppress inflammation in old donors^67^. NSAID works by inhibiting cyclooxygenase (COX) enzymes, which play a crucial role in the inflammatory process and oxidative stress. While preclinical models suggest that aspirin may reduce IRI-related inflammation and thrombosis in vascular transplant models^68^, studies in aged donor organs remain limited.

Statins, commonly known for their cholesterol-lowering effects, also have a significant anti-inflammatory property^69^. Statins inhibit the NF-kB pathway, a key regulator of inflammation. Statins also reduce the levels of C-reactive protein (CRP), a potent activator of complement system and inflammatory cytokines. Statins have also been brought forward as a potential treatment interfering with atherosclerotic inflammation and post-transplant vasculopathy. While data have not been univocally in support, a randomized clinical trial demonstrated that early initiation of simvastatin after heart transplantation significantly improved 8-year survival ameliorating transplant vasculopathy in the absence of adverse effects^70^. In contrast, renal transplant recipients did not seem to benefit from statin therapy, emphasizing on the complexity between organ specificities, quality and recipient characteristics^71^.

Vascular Endothelial Growth Factor a (VEGF-a) has been identified as a key player in organ rejuvenation, by promoting angiogenesis and reducing inflammation^72^. VEGF-A exerts its anti-inflammatory effects by reducing leukocyte adhesion, suppressing pro-inflammatory cytokine release, and enhancing endothelial barrier function. In a rat liver transplant model, delivery of a VEGF plasmid in combination with endothelial precursor cells ameliorated ischemia–reperfusion injury and enhanced angiogenesis, highlighting VEGF’s therapeutic potential in transplantation^73^. Additionally, many senolytic and senomorphic drugs also act as an anti-inflammatory agent due to their ability to reduce SASP factors.

### Mitochondrial modulators

Mitochondrial dysfunction is another major driver of organ age. This process is characterized by a compromised respiratory capacity, reduced mitochondrial membrane potential, and increased production of ROS^74^. These changes are significantly associated with accelerated aging and functional decline in older donor organs^75^. Mitochondrial modulators are therefore gaining attention for their potential to rejuvenate organs by enhancing mitochondrial function and reducing cellular damage (Fig. 3C).

Coenzyme Q10 (CoQ10), an essential component of the mitochondrial electron transport chain, has shown promise in enhancing mitochondrial function and reducing cellular damage in aged organs^76^. CoQ10 effectively reduces oxidative stress, mitochondrial damage, and inflammation during IRI thereby improving cardiac graft function in a murine heart transplantation model^77^.

Nicotinamide riboside (NR), a precursor of Nicotinamide adenine dinucleotide (NAD) is an essential nucleotide in the production of ATP from mitochondria^78^. NAD accepts electrons from a variety of sources and transfers them to complex I of the electron transport chain, ultimately resulting in the generation of ATP. NR supplementation has been shown to enhance mitochondrial function and biogenesis in skeletal muscles of old mice by increasing NAD levels^79^. Pyrroloquinoline Quinone (PQQ) is another agent with rejuvenating potential known for supporting the formation and function of mitochondria^80^. Previous studies have shown that PQQ improves long-term survival of adipocytes by alleviating hypoxic stress and promoting angiogenesis in early phase following transplantation^81^. Moreover, PQQ stimulates mitochondrial biogenesis in fibroblasts and hepatocytes via the SIRT1/PGC-1α pathway with preclinical research suggesting that it may slow down aging by enhancing mitochondrial function and reducing oxidative stress and inflammation.

### Anti-fibrotic agents (Fibrinolytics)

Fibrosis, a hallmark of aging, is characterized by the accumulation of extracellular matrix components, leading to tissue scarring and organ dysfunction. The leading causes of fibrosis in older organs correlate to SASP-induced chronic inflammation and decreased repair capacity. Clinically used anti-fibrotic agents including nintedanib and pirfenidone are designed to reduce the formation of fibrotic tissues. Notably, when utilizing older organs for transplantation it appears of critical relevance to identify drugs that effectively eliminate fibrotic tissues already formed in older organs. Although the experience of using fibrinolytic agents in organ transplantation is currently limited, there are some promising drugs that aim to not only reduce the formation of fibrosis but also target and potentially reverse existing fibrotic scars (Fig. 3D). In clinical kidney transplantation, early post-transplant administration of losartan significantly reduced interstitial fibrosis, improved graft structure, and decreased TGF-β1 and PAI-1 levels, both of which are central to fibrosis progression^82^. Additional clinical studies demonstrated less fibrosis and improved long-term graft function in patients receiving losartan early after transplantation^83^. The anti-fibrotic effect of losartan has also been clinically demonstrated in patients with idiopathic pulmonary fibrosis^84^.

Blebbistatin, a non-muscle myosin II inhibitor, has also shown significant potential in reducing fibrosis. This agent works by reducing the stiffness of fibrotic tissues, a process that in turn decreases the activation of myofibroblasts—cells that play a key role in the formation of fibrotic scars. Recently it has been shown that Blebbistatin significantly inhibits experimental liver fibrosis by reducing tissue stiffness and myofibroblast activation^85^.

Nattokinase, an enzyme derived from fermented soybeans, also has fibrinolytic properties and has been studied for its potential to degrade fibrin, thus ameliorating fibrosis^86^. Lumbrokinase, extracted from earthworms, has strong fibrinolytic activities and has been investigated for its ability to break down fibrin^87^. These enzymes can degrade fibrin and other extracellular matrix components, making them potential candidates for anti-fibrotic therapy.

## Biotechnological strategies of rejuvenation

### Stem cell therapy

Stem cell therapy has shown significant potential in treating age-related diseases by regenerating and repairing damaged tissues with the potential to repair failing organs and restore their function, potentially reducing the need for transplants^88^. Most of the stem cells are derived from bone marrow and fat tissues (mesenchymal stem cells, MSCs) or reprogrammed back to a pluripotent state through the induction of certain genes (induced pluripotent stem cells, iPSCs)^89^. To generate specific cell types (e.g., neurons, heart cells), stem cells are exposed to signaling molecules and growth factors that direct them to differentiate^90^. Once differentiated, the cells are purified to isolate the desired cell type for therapeutic or research purposes.

Stem cells secrete growth factors that promote angiogenesis, reduce inflammation, and prevent fibrosis, all common problems in old organs. Studies have shown that stem cells, particularly iPSCs, can improve heart function by regenerating damaged heart muscle tissue in old or failing hearts^91^. Early clinical trials in patients with heart disease have shown improved heart function after stem cell injections, suggesting a potential use for improving donor heart viability before transplantation^92^. MSCs have also been shown to improve the function of old kidneys and livers by reducing fibrosis and inflammation^93^. In animal studies, old kidneys treated with MSCs before transplantation demonstrated better graft function and lower rates of failure^94^.

Another critical advantage of stem cell therapy is its ability to modulate the immune system. Traditional immunosuppressive agents, though effective, come with significant long-term side consequences including increased risks for infections and malignancies. Stem cells, especially MSCs can help modulate the recipient’s immune system, reducing the likelihood of graft rejection and minimizing the need for potent immunosuppression^95^. They do so by the secretion of several factors including transforming growth factor-beta (TGF-β) and interleukin-10 (IL-10), all playing a key role in converting conventional T-cells into regulatory T-cells (Tregs). Kidney transplant recipients treated with MSCs required fewer immunosuppressive drugs while demonstrating reduced acute rejection rates and improved graft function^96^.

It is important to note that some studies have reported inconclusive results regarding stem cell viability, engraftment efficiency, and the long-term impact on graft survival, which currently limits the widespread clinical application of MSC-based therapies. Continued research is warranted to overcome these challenges, optimize delivery strategies, and better define the conditions under which stem cell therapies can consistently improve outcomes in the setting of older donor organs.

### Vaccine therapy

Recently, a vaccine therapy was introduced as a novel alternative to senolytic treatments, effectively eliminating senescent endothelial cells^97^. Glycoprotein non-metastatic melanoma protein B (GPNMB), a type I transmembrane protein expressed in various tissues, including vascular endothelial cells has been identified as a marker of cellular senescence. A peptide vaccine targeting the GPNMB protein was developed, inducing the production of antibodies that specifically recognize and bind to GPNMB-positive senescent cells. These antibodies tag senescent cells that are subsequently targeted and eliminated by immune cells. Experimentally, administration of this GPNMB-based vaccine in murine models elicited an antibody-dependent cellular cytotoxic response mediated by natural killer cells. Moreover, GPNMB-based vaccination protected mice against vascular plaque burden while extending lifespan in an accelerated aging model characterized by vascular pathology. Importantly, application of the GPNMB vaccine demonstrated superior outcomes compared to other senolytic treatments with a consistent reduction of senescent-like phenotypic changes while reducing off-target effects.

## Novel organ preservation methods

### Organ perfusion

Machine perfusion (MP), specifically normothermic perfusion, has emerged as an advanced technique to preserve, assess, and potentially rejuvenate organs ex-vivo prior to transplantation^98^. As the demand for transplantable organs rises, MP has become increasingly relevant due to its ability to maintain organ viability beyond the limitations of static cold storage, aspects that are especially relevant when considering organs from older and marginal donors. Unlike traditional static cold storage, MP allows organs to be supplied with oxygen, nutrients, and potentially various therapeutic agents, improving their viability and function.

Clinical evidence from heart, lung, kidney, and liver transplants strongly supports the efficacy of MP in enhancing the viability and function of donor organs, particularly those from older or marginal donors^99^. By minimizing cold ischemic time and maintaining near-physiological conditions, MP has been shown to reduce endothelial injury, attenuate inflammation, and improve early graft function. Moreover, preclinical studies have delineated how MP can improve organ quality and serve as a platform for testing therapeutic interventions^100^.

With particular relevance for the utilization of older donor organs, MP has the potential not only to provide an improved preservation technique but also to serve as a therapeutic platform—enabling the targeted delivery of therapeutic agents while minimizing off—target effects and enhancing localized treatment efficacy. Various interventions have been explored in preclinical models to enhance the therapeutic potential of MP^101^. Stem cell therapy, particularly with MSCs, has demonstrated reduced inflammation and fibrosis, along with improved renal function in aged donor kidneys during normothermic perfusion. Anti-inflammatory agents including corticosteroids and N–acetylcysteine have been shown to mitigate ischemia-reperfusion injury in liver and kidney models by decreasing cytokine release and oxidative stress. Vasodilators, including nitroglycerin and prostacyclin analogs, have also been used during MP to improve perfusion dynamics and endothelial function of aged organs in preclinical models^102^.

Recent advances have extended MP’s capabilities to include gene-based therapies. siRNA delivery during MP—targeting complement and Fas pathways—has successfully reduced IRI and improved graft function in rodent kidney and liver models^103^. Moreover, viral vector-mediated gene therapy including adeno-associated virus- based cardiac transgene delivery during normothermic perfusion has demonstrated stable, organ—specific expression with minimal off-target distribution in large animal heart transplant models^104^. These findings highlight MP’s versatility as a tool for both organ preservation and therapeutic innovation.

Normothermic Regional Perfusion (NRP) represents another novel technique^105^. The procedure involves restoring blood flow to donor organs in-situ using mechanical support, perfusing organs at normothermic conditions with oxygenated blood^106^. NRP can be delivered in as thoracic-abdominal (TA-NRP) – perfusing both thoracic and abdominal organs or with an abdominal approach (A-NRP), limiting the perfusion abdominal organs^107^. NRP is currently applied in the setting of DCD and may be particularly relevance for older DCD donors^108^. NRP also offers a unique opportunity to deliver rejuvenating therapies under physiological conditions, potentially enhancing organ recovery prior to transplantation. Unlike MP, which maintains organ perfusion ex-vivo, NRP allows for physiological restoration of organ function in-situ, potentially better mimicking natural conditions. This technique may result in more comprehensive organ assessment and recovery of function compared to MP.

### Subnormothermic preservation

Tissue handling and preservation conditions at the time of procurement play a critical role in determining post-transplant graft function. Among emerging approaches, static storage at 10 °C has gained significant attention as a superior alternative to traditional ice-cold (4 °C) storage. Early work a canine model demonstrated that lungs preserved at 10 °C maintained higher levels of ATP and superior function compared to organs stored at 4 °C in^109^. More recent studies have confirmed these findings showing that lungs stored at 10 °C preserved mitochondrial health resulting in improved function in both preclinical and clinical settings^110,111^. Additionally, 10 °C static preservation of porcine livers following circulatory death has been shown to improve biliary viability and reduce ischemia-reperfusion injury, highlighting the broader applicability of this approach beyond pulmonary grafts^112^. These findings underscore that even modest changes in preservation temperature can substantially influence organ metabolism, injury, and viability, and may provide an important adjunct to therapeutic strategies aimed at mitigating age-related vulnerability and ischemic damage.

### Heterochronic transplantation

Heterochronic transplantation (HCTx) characterizes an approach in which cells, tissues, or organs are transplanted in an age-mismatched fashion (young to old or old to young)^113^. This approach leverages the biological differences between young and old organs to delineate the influence of the cellular environment on organ rejuvenation or that of an organ to rejuvenate its environment. A common experimental model for studying HCTx is heterochronic parabiosis, a model in which the circulatory systems of a young and an old animal are surgically joined, allowing the two organisms to share blood-borne factors^114^. Through this model, researchers have observed that older animals often exhibit improved muscle repair, enhanced neurogenesis, and organ regeneration subsequent to the exposure to young blood, while young animals connected to older partners show reduced regenerative capacities and accelerated aging in certain tissues. HCTx suggests three key mechanisms that may carry rejuvenative/aging effects:

#### Circulating factors and secreted proteins

Certain proteins and signaling molecules in young organisms including GDF11 and other components related to the TGF-β signaling pathway, have been shown to play a critical role for rejuvenation^115^. GDF11 has the capacity to reverse some age-related cardiac and muscular decline in older animals. Additionally, factors including complement protein C1q have been identified as systemic agents that promote aging-related phenotypes by activating aging pathways that include Wnt signaling^116^.

#### Extracellular Vesicles (EVs)

EVs are small particles secreted by cells, containing proteins, mRNAs, miRNAs, and lipids affecting the functions of distant cells^117^. EVs from young cells are often enriched with factors that support stem cell function while inhibiting senescence. EVs from older cells, in contrast, carry signals that promote inflammation and cellular aging^118^. The presence of EVs from young cells in an older organism has been linked to rejuvenative effects, including enhanced regenerative capacities.

#### Direct cellular effects and stem cell integration

Transplanted young stem cells may physically integrate into recipient’s tissues, differentiating into functional cells^119^. In support, transplanted young hematopoietic stem cells have been shown to engraft into the bone marrow of older recipients, enhancing immune functions. Using a mouse model of heterotopic heart transplantation, we delineated that cardiac transplants rapidly adapt to the age of the recipient. Younger hearts grafted into older animals exhibit accelerated aging, while younger recipients promote the rejuvenation of grafted old hearts^120^.

**Fig. 4:**
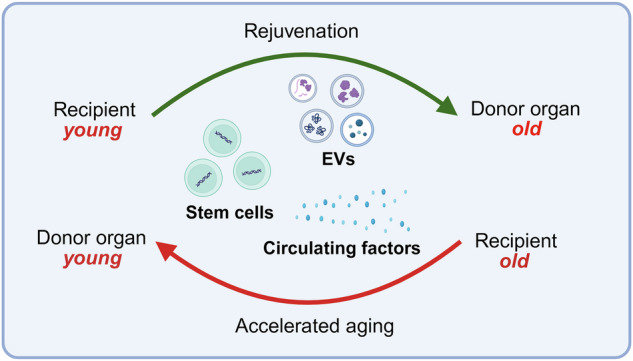
Heterochronic transplantation: Bidirectional effects of donor and recipient age in transplantation. In heterochronic (age-disparate) transplantation, the host environment of young recipients may ameliorate age-associated dysfunctions of older, promoting rejuvenation via the exchange of extracellular vesicles, stem cells, and other yet unknown circulating elements. Conversely, when young organ are transplanted into old recipients, systemic responses may accelerate aging processes in the transplanted organ, highlighting the critical role of systemic environment in organ rejuvenation. Effects of donor age may also have a systemic impact on recipient’s age. *Created in BioRender. Kayumov, M. (2025)* https://BioRender.com/m23u7ro.

Thus, HCTx holds substantial therapeutic potential when transplanting older organs. Based on preclinical research findings, one could envision that older donor organs, when transplanted into younger recipients, may undergo rejuvenation and assimilate to the physiological environment of younger recipients, aspects with relevance for organ allocation and utilization (Fig. 4).

## Clinical applications and evidence

Although rejuvenation strategies for donor organs are still primarily explored in experimental models, several clinical approaches have shown promising results in improving the viability and function of organs prior to transplantation. For instance, studies in liver transplantation have shown that N-acetylcysteine, when delivered during machine perfusion, may ameliorate reperfusion injury and improve post-transplant graft function^121^. The use of statins in kidney transplantation has been shown to reduce inflammation while improving outcomes when administered before or during machine perfusion^122,123^. In addition, MSCs were used in clinical studies to ameliorate inflammation and promote repair in donor organs. Early-stage clinical trials have tested the infusion of MSCs during kidney and liver transplantations, showing reduced rates of rejection and improved early graft function^124^. Moreover, MSCs have been tested to repair damage after myocardial infarction, with early results suggesting improved myocardial function^125^. However, variability in MSC sources, dosing, timing, and delivery routes continues to limit reproducibility and generalizability of reported outcomes.

Senolytics, although still in early stages of clinical trials, offer another promising avenue. Previous studies have shown that the use of senolytic agents including D&Q can reduce senescent cell populations in older organs, potentially improving graft function and transplant outcome^28,29^. Our own work has demonstrated that D&Q effectively clear senescent cells from old cardiac allografts while reducing cell-free mitochondrial DNA, a key driver of age-associated inflammation. By targeting cellular senescence, these compounds mitigate immune responses of the recipient and significantly improve the survival of older donor organs in transplantation. While promising, clinical trials using senolytics in transplantation are currently lacking, and safety profiles must be established due to concerns over off-target toxicity, especially in immunocompromised patients.

Other studies have explored the use of growth factors including VEGF to enhance organ repair^126^. For instance, perfusing kidneys with growth factors during machine perfusion has shown improved graft function by promoting angiogenesis and tissue repair^126^.

While clinical applications of rejuvenating strategies are still evolving, the integration of machine perfusion and therapies aimed at reducing senescent cell burden and IRI has already made a notable impact. Emerging treatments including senotherapeutics, stem cell therapy, and targeted pharmacological agents continue to hold great promise for improving the quality of donor organs, particularly those from older or marginal donors.

However, translating these advances into routine clinical practice remains challenging. Key obstacles include variability in donor characteristics and perfusion protocols, regulatory hurdles associated with novel therapeutics, and a lack of robust long-term outcome data, particularly for grafts treated with rejuvenation agents.

## Future directions and challenges

Organ rejuvenative strategies including organ-specific approaches will most likely see increased clinical application in the near future. Strategies may include agents improving mitochondrial function, reducing the burden of senescent cells and oxidative stress while enhancing tissue repair. The use of MP platforms especially normothermic perfusion provides a promising environment not only for preservation but also for the delivery of rejuvenative therapies. Notably, prolonged normothermic perfusion durations may enable more effective implementation of pharmacological or cellular interventions, allowing for real-time assessment and optimization of organ quality before implantation.

While some preclinical studies have explored the concept of rejuvenation through exposure to young plasma components, the idea of using “young blood” in isolation remains speculative. Therefore, future research should aim to identify and isolate specific rejuvenating factors from young systemic environments and evaluate their efficacy when delivered via machine perfusion in a controlled, targeted manner.

Another promising approach could be the application of a two-phase rejuvenation strategy. At least in theory, in the pre-transplant phase, donor organs could be treated with rejuvenating “cocktails” during machine perfusion. This approach may target aging processes including cellular senescence, inflammation, and oxidative stress to enhance organ function and viability. After transplantation, stem cell therapies may be administered to promote tissue repair while modulating immune responses that may reduce the immunogenicity of older organs, thus ameliorating the rates of acute and chronic rejections.

To facilitate more clinical implementation, future research should prioritize multicenter randomized trials to validate early findings, longitudinal studies to assess graft longevity and post-transplant complications, the development of standardized protocols for therapeutic delivery via machine perfusion, and the identification of reliable biomarkers to monitor treatment efficacy in real-time. On the way to advancing rejuvenation strategies significant hurdles may be best addressed through collaborative efforts between researchers, clinicians, and industry.

## Conclusion

The use of older donor organs presents challenges, particularly related to an increased vulnerability to IRI, higher rates of delayed graft function, and amplified and compromised organ quality. Rejuvenative strategies hold significant promises for expanding the donor organ pool by improving the quality and viability of these older organs. By integrating machine perfusion technologies, senotherapeutics, mitochondrial modulators, heterochronic transplantation and stem cell therapies, it may be feasible to mitigate the effects of aging, functional decline, augmented immunogenicity, and the detrimental consequences of IRI in older organs.
